# Supplementary material for: Genetic evidence for a regulated cysteine protease catalytic triad in LegA7, a Legionella pneumophila protein that impinges on a stress response pathway
Source: mSphere. 2024 Aug 21;9(9):e00222-24. doi: 10.1128/msphere.00222-24 (PMC11423584; doi:10.1128/msphere.00222-24)
Supplement: Supplemental figures — Figures S1 to S3. [file msphere.00222-24-s0001.pdf]

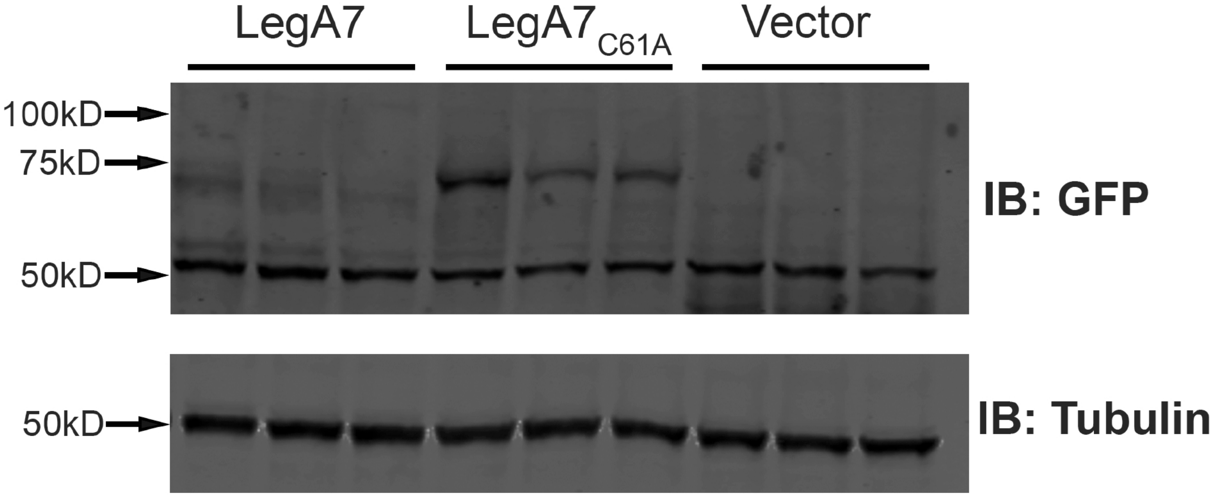

Supplemental Fig. S1. Mutation in putative catalytic residue C61 reduces degradation of LegA7. HEK293T were transfected with the noted plasmids and 40 hrs after transfection, the cultures were extracted, gel fractionated and probed with noted antibodies (Materials and Methods). Shown are three independent transfections with each plasmid. LegA7: GFP-LegA7 derivative described in text (Materials and Methods); $LegA7_{C61A}$: GFP-LegA7 having Cys->Ala mutation in predicted catalytic residue C61.

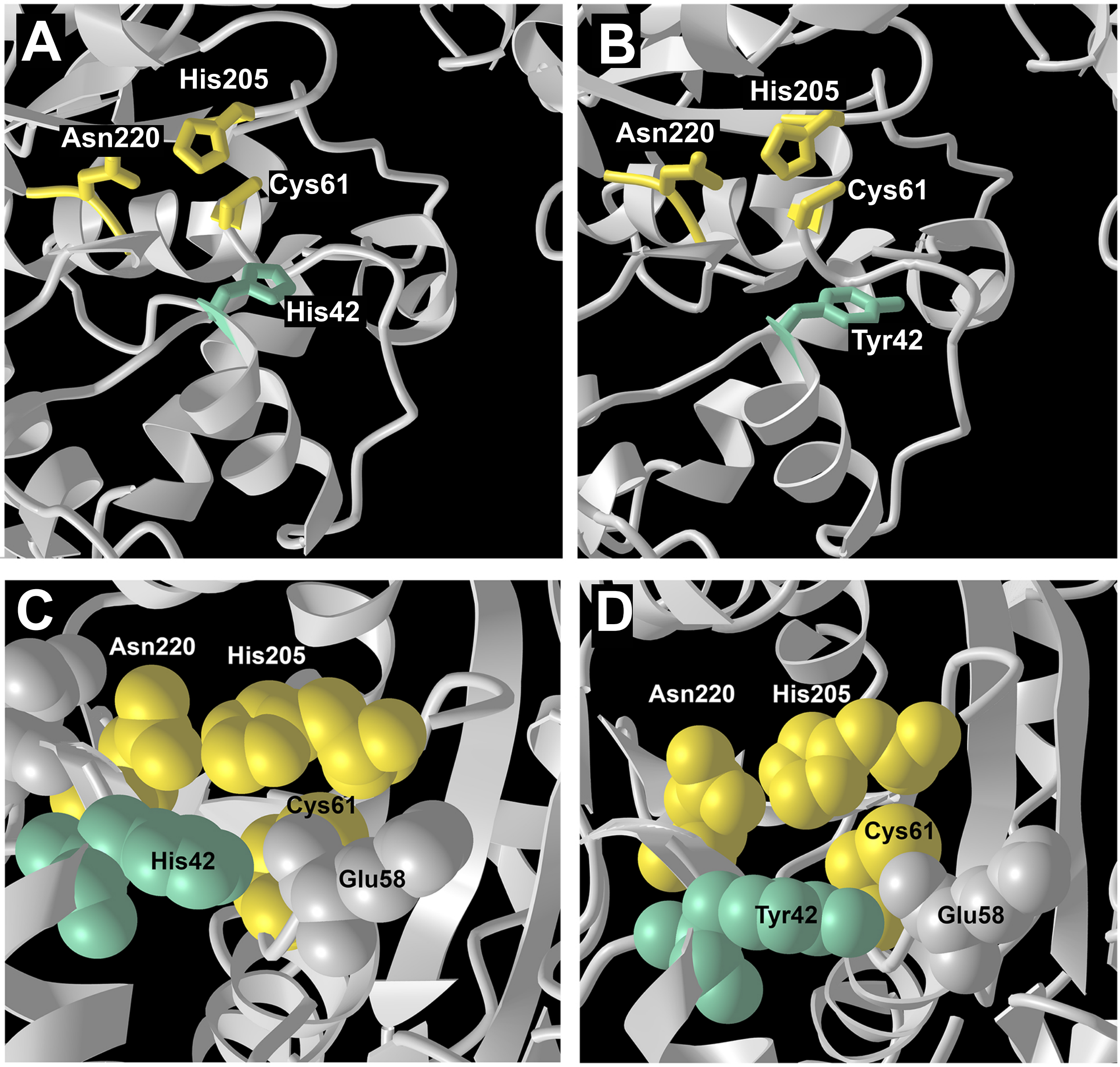

Fig. S2. Modeling of H42Y mutation places the Tyr sidechain proximal to the LegA7 cysteine protease catalytic site. Shown is the LegA7 catalytic triad (Cys61, His205, Asn220) displayed as yellow sidechains, with the nearby His42 (green) or the Tyr42 replacement. (A, B) Stick figures of relevant sidechains for WT (A) and Tyr42 mutation (B). (C,D) Space filling models of relevant sidechains for WT (C) and Tyr42 mutation (D).

> Δank-1(331-454)_deletion endpoints (sense)
CGACAACGACTTAGTGATGATGAGGAGAGATTGGAAAAAGAA
R Q R L S D D E E R L E K E

>Δank-1(331-454)_translation
MIYVLICIMLVCGLVVYQLMPKLLSYSTSLISKKQDTSELTHDGITNVLTQLGHPKFEGVCYGFTLNWALAVAQGKESFFYRQLH
HLRTHQFGLPETLQQIKEKKERNQSLSKDEKIIETLPQLGKKICIAQDPLQYKEKYKKLVWQPDINSILKAINADSSVAKHIFYK
THSFLNQDEATEYLELLKRTGIREDVAVIISTADHAMGFKLAGNVWRFININDLYQQDKNKPYFEFSSRNLVKELYRVCAENLQG
SRLTVNTDFVSVNPEEKLSRALQNLFPVFPVRTKTSYPERLAFFSMAATQGDMNSVKKCIHSGWSIFSRQRLSDDSPILTAIYLG
RRDVVRAMLSTSRHRVNQKRKSDSSTLLHIACRYGGSGIVEDLLNIRGIKIDPRDSKGRTPLMYACKKSVVTEDRKLFNLLFAKG
ASLSIKDNDGLTALDHALKNEHTLAIQMI**EERLEKEACAQENSTSRRFKFSETKGTLFQRGVKTISYQSQQPRFGMK**

> Δank-2(290-454)_ deletion endpoints(sense)
TTTCCAGTCAGAACCAAAGAGGAGAGATTGGAAAAAGAA
F P V R T K E E R L E K E

> Δank-2(290-454)_translation
MIYVLICIMLVCGLVVYQLMPKLLSYSTSLISKKQDTSELTHDGITNVLTQLGHPKFEGVCYGFTLNWALAVAQGKESFFYRQLH
HLRTHQFGLPETLQQIKEKKERNQSLSKDEKIIETLPQLGKKICIAQDPLQYKEKYKKLVWQPDINSILKAINADSSVAKHIFYK
THSFLNQDEATEYLELLKRTGIREDVAVIISTADHAMGFKLAGNVWRFININDLYQQDKNKPYFEFSSRNLVKELYRVCAENLQG
SRLTVNTDFVSVNPEEKLSRALQNLFPVFPVRTKTSYPERLAFFSMAATQGDMNSVKKCIHSGWSIFSRQRLSDDSPILTAIYLG
RRDVVRAMLSTSRHRVNQKRKSDSSTLLHIACRYGGSGIVEDLLNIRGIKIDPRDSKGRTPLMYACKKSVVTEDRKLFNLLFAKG
ASLSIKDNDGLTALDHALKNEHTLAIQMI**EERLEKEACAQENSTSRRFKFSETKGTLFQRGVKTISYQSQQPRFGMK**

> ΔN-ank-1(264-361)_deletion endpoints(sense)
CGATTGACCGTGAATACCGATAGCGACTCTTCTACTTTGCTC
R L T V N T D S D S S T L L

> ΔN-ank-1(264-361)_translation
MIYVLICIMLVCGLVVYQLMPKLLSYSTSLISKKQDTSELTHDGITNVLTQLGHPKFEGVCYGFTLNWALAVAQGKESFFYRQLH
HLRTHQFGLPETLQQIKEKKERNQSLSKDEKIIETLPQLGKKICIAQDPLQYKEKYKKLVWQPDINSILKAINADSSVAKHIFYK
THSFLNQDEATEYLELLKRTGIREDVAVIISTADHAMGFKLAGNVWRFININDLYQQDKNKPYFEFSSRNLVKELYRVCAENLQG
SRLTVNTDFVSVNPEEKLSRALQNLFPVFPVRTKTSYPERLAFFSMAATQGDMNSVKKCIHSGWSIFSRQRLSDDSPILTAIYLG
RRDVVRAMLSTSRHRVNQKRK**SDSSTLLHIACRYGGSGIVEDLLNIRGIKIDPRDSKGRTPLMYACKKSVVTEDRKLFNLLFAKG**
ASLSIKDNDGLTALDHALKNEHTLAIQMIEERLEKEACAQENSTSRRFKFSETKGTLFQRGVKTISYQSQQPRFGMK

> ΔN-ank-2(290-361)_deletion endpoints(sense)
GTGTTTCCAGTCAGAACCAAAAGCGACTCTTCTACTTTGCTC
V F P V R T K S D S S T L L

> ΔN-ank-2(290-361)_translation
MIYVLICIMLVCGLVVYQLMPKLLSYSTSLISKKQDTSELTHDGITNVLTQLGHPKFEGVCYGFTLNWALAVAQGKESFFYRQLH
HLRTHQFGLPETLQQIKEKKERNQSLSKDEKIIETLPQLGKKICIAQDPLQYKEKYKKLVWQPDINSILKAINADSSVAKHIFYK
THSFLNQDEATEYLELLKRTGIREDVAVIISTADHAMGFKLAGNVWRFININDLYQQDKNKPYFEFSSRNLVKELYRVCAENLQG
SRLTVNTDFVSVNPEEKLSRALQNLFPVFPVRTKTSYPERLAFFSMAATQGDMNSVKKCIHSGWSIFSRQRLSDDSPILTAIYLG
RRDVVRAMLSTSRHRVNQKRK**SDSSTLLHIACRYGGSGIVEDLLNIRGIKIDPRDSKGRTPLMYACKKSVVTEDRKLFNLLFAKG**
ASLSIKDNDGLTALDHALKNEHTLAIQMIEERLEKEACAQENSTSRRFKFSETKGTLFQRGVKTISYQSQQPRFGMK

Supplemental Figure S3. Endpoints of deletions described in Fig. 6. Shown are the DNA sequence endpoints of each deletion mutant described in Fig. 6 and the predicted amino acid sequence of each derivative, based on whole plasmid sequencing (Materials and Methods). Red: nucleotide sequence of 3' end of deletion. Black Bold: amino acid sequences remaining in derivative. Gray: amino acid sequences missing.
